# Asynchrony between Host Plant and Insects-Defoliator within a Tritrophic System: The Role of Herbivore Innate Immunity

**DOI:** 10.1371/journal.pone.0130988

**Published:** 2015-06-26

**Authors:** Vyacheslav V. Martemyanov, Sergey V. Pavlushin, Ivan M. Dubovskiy, Yuliya V. Yushkova, Sergey V. Morosov, Elena I. Chernyak, Vadim M. Efimov, Teija Ruuhola, Victor V. Glupov

**Affiliations:** 1 Laboratory of Insect Pathology, Institute of Systematics and Ecology of Animals SB RAS, Novosibirsk, Russia; 2 Biological Institute, National Research Tomsk State University, Tomsk, Russia; 3 Laboratory of Ecological Research and Chromatographic Analysis, Novosibirsk Institute of Organic Chemistry SB RAS, Novosibirsk, Russia; 4 Laboratory of Molecular-Genetic Systems, Institute of Cytology and Genetics SB RAS, Novosibirsk, Russia; 5 Department of Biology, University of Eastern Finland, Joensuu, Finland; Institute of Plant Physiology and Ecology, CHINA

## Abstract

The effects of asynchrony in the phenology of spring-feeding insect-defoliators and their host plants on insects’ fitness, as well as the importance of this effect for the population dynamics of outbreaking species of insects, is a widespread and well-documented phenomenon. However, the spreading of this phenomenon through the food chain, and especially those mechanisms operating this spreading, are still unclear. In this paper, we study the effect of seasonally declined leafquality (estimated in terms of phenolics and nitrogen content) on herbivore fitness, immune parameters and resistance against pathogen by using the silver birch *Betula pendula*—gypsy moth *Lymantria dispar*—nucleopolyhedrovirus as the tritrophic system. We show that a phenological mismatch induced by the delay in the emergence of gypsy moth larvae and following feeding on mature leaves has negative effects on the female pupal weight, on the rate of larval development and on the activity of phenoloxidase in the plasma of haemolymph. In addition, the larval susceptibility to exogenous nucleopolyhydrovirus infection as well as covert virus activation were both enhanced due to the phenological mismatch. The observed effects of phenological mismatch on insect-baculovirus interaction may partially explain the strong and fast fluctuations in the population dynamics of the gypsy moth that is often observed in the studied part of the defoliator area. This study also reveals some indirect mechanisms of effect related to host plant quality, which operate through the insect innate immune status and affect resistance to both exogenous and endogenous virus.

## Introduction

Between the emergence of herbivorous insects and the budding of their host plants, the importance of phenological synchrony for the population dynamics of spring-feeding insects is well-documented [1–5]. The hatching of larvae before trees burst into bud may lead to their starvation, whereas the quality of foliage is poor if the larvae hatch too late [4]. The latter phenomenon results from a rapid decrease in the protein and water content of tree leaves and a concomitant increase in leaf toughness with leaf maturation [2, 6–7]. Also, the concentration of secondary compounds in tree leaves, which plays an important role in plant defence, has been shown to change markedly with leaf development [6–9].

Meanwhile, the studies of the last century clearly show that variations in the quality of consumed leaves (caused by the effect of different factors) modify the interactions between herbivorous insects and their parasites (reviewed in [10]), indirectly affecting the population dynamics of the folivores. However, there are only a few studies addressing the role of the phenologically associated decline in host plant quality in the modification of interactions between different levels of consumers within the same food chain [11–13]. Similarly, few studies attempt to even peculate on how the quality of the primary producer is delivered to different levels of consumers [14].

It is well known that insects possess effective barriers, such as an innate immunity, effectively protecting them against attack from parasites. The innate immunity of insects consists of the structures executing barrier functions (cuticle, peritrophic envelope), and the mechanisms of cellular and humoral immunity (e.g. encapsulation, phagocytosis, antimicrobial peptides, etc.) [15]. The encapsulation of the invader followed by melanisation is an effective response of insects to parasitoids or pathogens [16]. In cellular encapsulation, the formation of capsules requires the aggregation of plasmatocytes and granulocytes around the invader [17]. The phenoloxidase (PO) cascade takes part in the melanisation of haemocytes attached to the surface of the parasite [18]. These parameters of innate immunity possess a wide norm of reaction and are modified by different environmental factors including the quality of consumed food (i.e. [19–24]). Thus, based on these arguments, we hypothesise that asynchrony-driven decline in the quality of consumed leaves may modify the interactions between insects-defoliators and their parasites through the host immune function changes that have not been studied up until now.

In this study, we used a well-known species, namely the gypsy moth *Lymantria dispar* L. (Lepidoptera: Lymantriidae). The gypsy moth is a typical eruptive, economically important species, belonging to the spring-feeding guild and inhabiting the temperate forests of Eurasia and North America. The outbreaks of *L*. *dispar*, accompanied by severe host plant defoliation, occur in many parts of its area in Eurasia [25–26] as well as in North America [27]. The successful hatching of gypsy moth larvae in synchrony with the budding of the main host plant species is essential for the successful development of insects, whereas mature leaves are inferior food for them [2, 13]. Moreover, it was also shown that the asynchronous hatching of *L*. *dispar* affects the larval mortality rate induced by natural parasites [13], or may even change the interactions between insect density and parasite-induced levels of insect mortality [12]. However, the importance of insect immune reactions (which most probably are affected by maturing foliage) for these interactions remains completely unknown.

In the present study, we employed the approach of measuring a host’s baseline innate immunity traits (phenoloxidase activity, encapsulation response, total haemocyte count) as the predictor of the level of resistance of *L*. *dispar* to the most important pathogens infecting the larval stage–nucleopolyhedrovirus under the effect of phenological mismatch. The *Lymantria dispar* multinucleopolyhedrovirus (LdMNPV, Baculoviridae) is a specialized virus inducing natural epizootics in gypsy moth populations and is widely used to control *L*. *dispar* in many countries [28–29]. To substantiate the potential resistance of larvae against the virus, we estimated the level of its actual resistance to the baculovirus. As with many baculoviruses, LdMNPV possesses the ability to stay inside the host body without symptomatic manifestations and can transfer from parental to filial generations by means of transovarial transmission [30–31]. In turn, some stressors might induce the activation of a covert infection to an overt (symptomatic) form. Therefore, we also estimated the level of covert-virus activation induced by the phenologically-assisted decline of host plant quality and linked it to the insect immunity status.

Thus, in the present study we addressed the following main question: how does feeding by phenologically different host plants affect the fitness, immune parameters and the resistance of *L*. *dispar* larvae to baculovirus infection?

## Materials and Methods

### Study site and species

The experiments were carried out in the summers of 2011 and 2012 at the Karasuk Research Station of the Institute of Systematics and Ecology of Animals Siberian Branch RAS, Western Siberia, Russia (53°42′N 77°45′E). In these experiments, young artificially planted *B*. *pendula* trees (~10 m tall) were used as the host plant and the primary producer. The trees were not noticeably attacked by wild defoliators during the six years prior to the experiments and the natural mean density of gypsy moth egg masses ranged from 0 to 0.05 egg masses per tree. Gypsy moth eggs for the current study were collected in the autumns of 2010 and 2011 from birch trees in the pest outbreak areas (Trans-Ural, Russia), where the moth populations were at their increase (pre-peak) phase of population cycle. Eggs were stored in a refrigerator at 4°C during the subsequent winter and used to provide a stock of experimental animals in the experiments of the following spring.

No permits for a field collection were required for this study, since the national forests in Russia are freely accessible. No protected species were sampled. Birch trees used for the feeding of insects in this study were grown on the territory owned by the Russian Academy of Sciences.

### General experimental design

**Fig 1:**
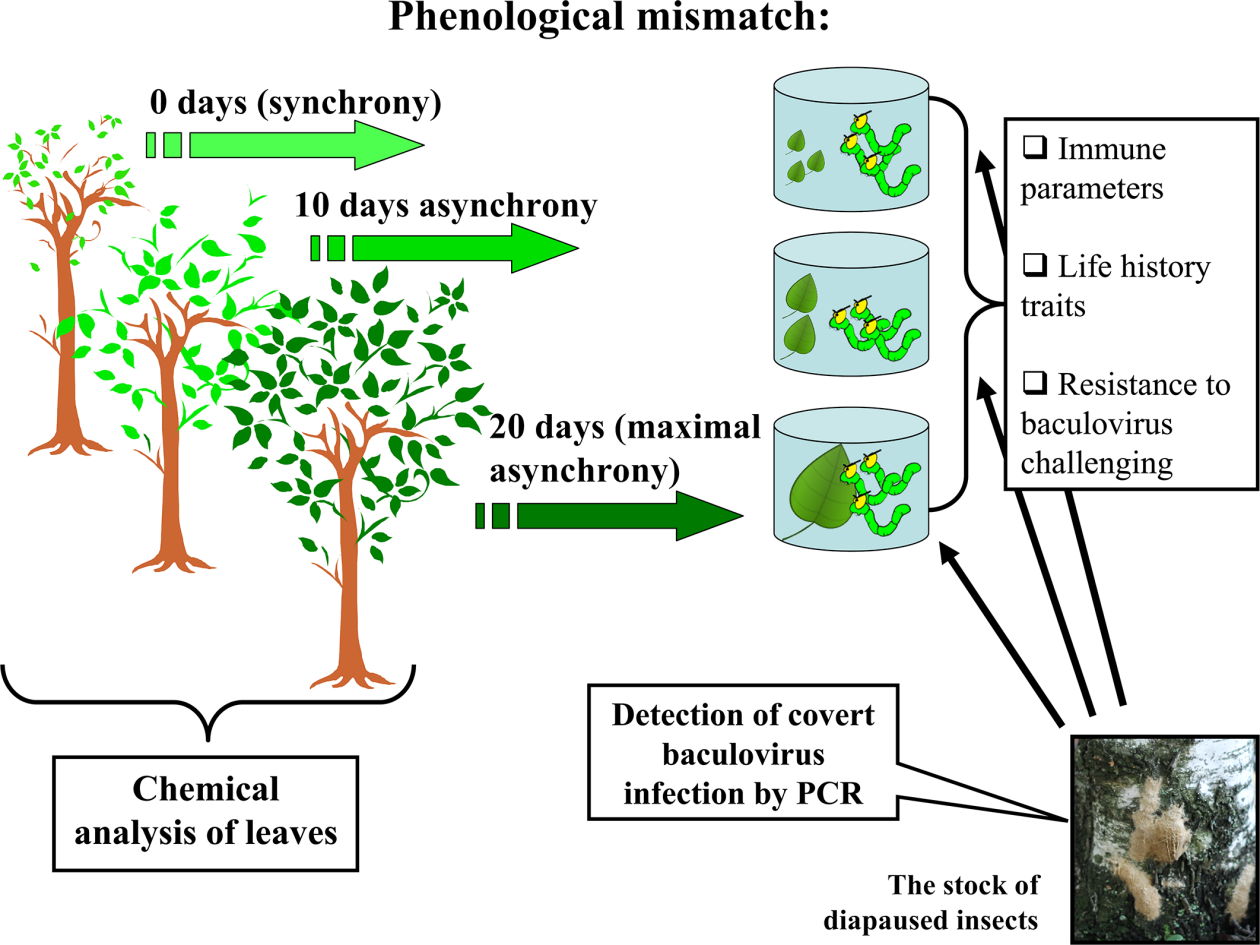
The scheme of experimental design. Five and fifteen days mismatches were excluded from the figure to make it more accessible.

In order to simulate the mismatch between the development of *L*. *dispar* larvae and *B*. *pendula* foliage, we modified the hatching time of the larvae. The general scheme of experimental design is presented in the scheme (Fig 1). The rearing of hatched larvae was performed in the laboratory on natural leaves of *B*. *pendula* at 22±1°C under a regime of natural daylight. For this experiment we used the mixed progeny of ca.200 females as the stock (Fig 1). To exclude the maternal effect, we cleaned the eggs of fluff following the insects’ wintering period and carefully randomized the eggs. The hatching of the larvae occurred within three days of pulling the eggs out of the refrigerator. Since there is a high variability in the hatching of larvae (even of larvae within same clutch) we conducted the larvae emergence under 28°C to facilitate the hatching process (i.e. so larvae bursted forth forcefully) and used only those larvae which emerged within 72 hours, and not any later. To feed the larvae we deliberately chose five trees of similar phenological states in order to minimize the variation in the budding between replicates. We used the following range of phenological mismatches between birch and gypsy moth (the length and width of *B*. *pendula* leaves is given in parenthesis ± SE): “0 days of mismatch” (synchronization point), hatching of larvae was synchronized with the budding of experimental trees (2.20±0.24 cm, 2.01±0.16 cm); “5 days of mismatch”—larvae were hatched five days after the synchronization point (2.81±0.18 cm, 2.55±0.20 cm); “10 days of mismatch” (3.90±0.10 cm, 3.26±0.03 cm), “15 days of mismatch” (4.50±0.11 cm, 3.91±0.18 cm), and “20 days of mismatch” (5.02±0.22 cm, 4.46±0.30 cm)—larvae hatched 10, 15 and 20 days after the synchronization point, respectively (Fig 1).

In the first year (2011), we reared the newly hatched first instar larvae on leaves collected from experimental trees until the larvae pupated (see [20]) in order to investigate the effects of asynchrony on the fitness parameters of insects. The random sampling of eggs was taken from the stock cohort of eggs before hatching so that an estimation of the percentage of virus-carrying insects (see below) could also be made. A cohort of 20 newly hatched larvae, maintained in a 5 L container, was used per each replicate (experimental tree) within each variant (days of phenological mismatch). The freshly cut leaves were washed with distilled water to exclude the environmental persistence of baculovirus. Then the leaves were put into 1.5 mL tubes filled with water and sealed with parafilm to maintain the cell turgor of the detached leaves. Prior to hatching, the eggs were surface-sterilized with 1% of sodium hypochlorite to prevent the transovum transmission of LdMNPV (i.e. through the surface contamination of eggs inside the females’ reproductive tract during oviposition) or the accidental contamination of eggs in the forest or in the laboratory. Thus, all the insects reared in the experiments might possibly contain only a transovarially transmitted virus, i.e. a virus located within the eggs. To control the effect of the prolonged diapause of eggs kept in a refrigerator (i.e. from 5 to 20 days for treated cohorts as compared with a synchronized cohort), we estimated the level of larvae hatching. This parameter varied between 82–90% with no statistically significant differences between the different levels of mismatch.

We repeated the above described procedure in the summer of 2012 to estimate the effects of asynchrony on the immune parameters of the gypsy moth (20 larvae per replicate) as well as on antiviral resistance (see below). However, in 2012, we used another set of *B*. *pendula* trees (five for immunity assay and three for virus inoculation assay) from the same plot to exclude the possible effects of artificial defoliation from the previous experiment.

Based on this experimental design there were, in theory, two options relating to the mode of action of phenological mismatch on insects: firstly, the variation of leaf quality during its maturation; and secondly, the variation in duration of eggs diapause. However, we point to the insignificant effect of the second option for the following reasons:

1. *L*. *dispar*, even after its hatching, is well adapted to such unfavourable conditions as starvation when the temperature is below 10°C [32], i.e. in our case, when eggs underwent diapause at 4°C the effect of prolongation was minimal;
2. The emergence rate of larvae was the same in all mismatched groups (see above);
3. The range of studied mismatches is consistent with the natural variation of *L*. *dispar* larvae hatching in the forests of the Siberia region (Martemyanov V.V., personal observation).

Thus, we believe that the main trigger driving the features of insects under studied conditions was the dynamics of the quality of consumed leaves during their maturation but not (or at least to a much less degree) the delay of diapause in insects groups. We will focus more on this statement in our discussion.

### Gypsy moth fitness traits

In the first growing season of 2011, we recorded the larval development time, the pupal weight, the overall mortality rate of insects [22] and a specific mortality rate as induced by activated covert LdMNPV. For the latter procedure, the insect cadavers were analyzed by light microscopy (axioskop 40, Carl Zess, Germany) to reveal viral occlusion bodies (OB). We also measured the initial rate of larvae that were harboring a covert virus by PCR (see below).

### Gypsy moth immune traits

Immune assays were conducted when larvae reached their fourth instar. Twenty larvae per tree per mismatch point were used. We individually estimated the encapsulation rate of nylon monofilament inserted into the hemocoel of larvae, the phenoloxidase activity in the plasma of haemolymph, and the total hemocyte count (THC) in haemolymph according to [22–23]. A detailed description of the procedures is presented in S1 Text. The encapsulation rate was measured as the degree of melanisation of a nylon monofilament implant inserted into the hemocoel of the larvae. This is one of the routine methods used to measure the encapsulation rate in the haemolymph of insects [20, 22–23, 33]. The implant was incubated for 3 h in larvae hemocoel after which the implant was removed and photographed as a black-and-white image from three different angles. The degree of the melanisation was quantified using Image Pro software (National Institute of Mental Health, Besheda, MD, USA) by measuring the colouration—the grey value (g.v.)—of all areas on each implant and then comparing these values with that of an unused implant. PO activity was measured with a plate reader (PowerWave HT, BioTek, USA) at 490 nm using L-DOPA (2 mg mL^-1^) as a substrate. Data are presented as units of transmission density (ΔA) of the incubation mixture during reaction per 1 min per 1 μL of plasma or per 1 min per 1 mg of protein. Haemolymph protein levels were measured according to [34] with a standard curve created from a bovine serum albumin standard. THC was measured by light microscopy (Axioskop 40, Carl Zess, Germany) with the help of a hemocytometer and was recorded as the number of hemocytes per 1 mL of haemolymph. All analysed larvae survived after measurement and were reared individually to adulthood for sex identification.

### The susceptibility of *L*. *dispar* to LdMNPV

For the testing of the susceptibility of larvae to exogenous virus in 2012, the inoculation was carried out using two concentrations of LdMNPV: a low concentration (10^4^ OBs mL^-1^ of suspension) and a medium concentration (10^6^ OBs mL^-1^ of suspension). One control group without a viral inoculation (i.e.treated with distilled water) was used per tree per each variant (days of mismatch). **We used 50 larvae per mismatch point, per concentration/control, per tree**. The inoculation was conducted perorally, according to [22]. The detailed technique of viral inoculation is presented in S1 Text. All experimental eggs were surface-sterilized as described above. We excluded the fifth- and the fifteenth-day variants of asynchrony, i.e. we had three asynchrony-treatment groups: 0, 10 and 20 days of asynchrony. Dead larvae were counted and analysed with a light microscope (axioskop 40, Carl Zess, Germany) to identify the baculoviral pathogenesis. We did not detect dead larvae caused by LdMNPV in any control variants, so we did not include the control cases in the presenting of the data.

### PCR analysis of *L*. *dispar* eggs for detecting of covert LdMNPV in stock population

We took 150 eggs from the stock cohort of eggs to identify the percentage of infected *L*. *dispar* individuals by covert baculovirus in the population. The eggs were sterilized by UV irradiation according to [35]. We used a different method of surface sterilization to serve as a comparison with the above described sterilization since we needed to prevent the contamination of the surface of the eggs by viral DNA (not only biological activity of virus). After sterilization, total DNA was extracted from individually homogenized eggs using phenol:chloroform:isoamyloalcohol (25:24:1) mixture, precipitated by isopropanol, then ethanol-washed, dried and re-suspended in molecular grade water [36]. Regions of polyhedrin genes were amplified and the amplicons were separated in 1% agarose gel electrophoresis in 1x TAE buffer. The detailed protocol is presented in the S1 Text. The low limit of reproducible detection was determined as the amount of DNA, isolated from virions of two viral polyhedrons. The results are presented as a percentage of PCR-positive (i.e. virus-containing) eggs.

### Chemical analysis of silver birch leaves

The leaves were collected in 2011 from the crowns of each of five experimental trees. Collections were made on five occasions in accordance with each mismatch date. Leaves were transported from the plot to the research station on ice, frozen at -20°C, and delivered frozen to the institute for freeze-drying.

Phenolic compounds (flavonoids and cinnamic acids) were extracted from the dried leaves by heating under reflux with ethanol: water (7:3, v/v) [37]. The extracts were then analysed using liquid chromatography instruments (Agilent 1200-system Agilent Technologies) supplied with a diode-array (DAD) and mass spectrometry micrOTOF-Q (Bruker) detectors (S1 Text). The identification of compounds was based on comparisons of the MS and UV data with the HPLC retention times (TR) [38]. The quantification of the flavonoids, 3,4'-dihydroxypropiophenone-3-β-D- glucopyranoside (DHPPG) and cinnamic acids in the leaves’ extracts was performed with the use of standard compounds rutin (Chemapol), quercetin (Sigma) and chlorogenic acid (Sigma).

We did not measure the content of either hydrolysable or condensed tannins in birch foliage, the dynamics of which are strongly determined by the maturation of leaves in deciduous trees [6, 9]. According to recent studies, the variation in the concentrations of both types of tannins in the diet did not affect the performance of those *L*. *dispar* reared on either *Quercus rubra* L. or *Acer saccharum* Marsh. [39] or on the hybrid poplar *Populus tremula* x P. alba [40], as opposed to other Lepidopteran species [41–43]. Moreover, according to [44], leaves of the *B*. *pendula* contain a low concentration of hydrolysable tannins, which might possess high oxidative activity (i.e. [45]).

The nitrogen content of leaves was determined using a CHNS analyzer EURO EA-3000 [46].

### Statistical analysis

We used regression analysis to estimate the effect of time on the concentrations of phenolics and nitrogen in the *B*. *pendula* leaves in spring since visually most of dynamics were close to the line. The effects of mismatch on the pupal weight, development time, and immune parameters were tested by mixed model analysis (SPSS 19.0 for Windows) wherein tree, tree*sex, tree*treatment and tree*sex*treatment were handled as random factors; sex, treatment and interaction were handled as fixed factors. We used the restricted maximum likelihood method for the estimation of the parameters and the type III sum of squares was chosen [22]. The larval development rate, PO activity and THC were log10-transformed prior to the analyses to meet the assumptions of the parametric tests. Results are presented as back-transformed data. To study the effects of the treatment and sex in more detail, pairwise contrasts were tested with the LSD-method. PO activity data was also tested vs. asynchrony value (days of mismatch) with regression analysis. We used one-way ANOVA to calculate the insect mortality rate data without infection (the experiment of 2011) and two-way ANOVA with the post-hoc Fisher LSD procedure to calculate the data of the mortality rate of infected insects (i.e. the experiment of 2012). Prior to analysis, all data in percentages were the arcsine of the square root-transformed.

## Results

### The effects of leaf maturation on the chemistry of silver birch leaves

**Fig 2:**
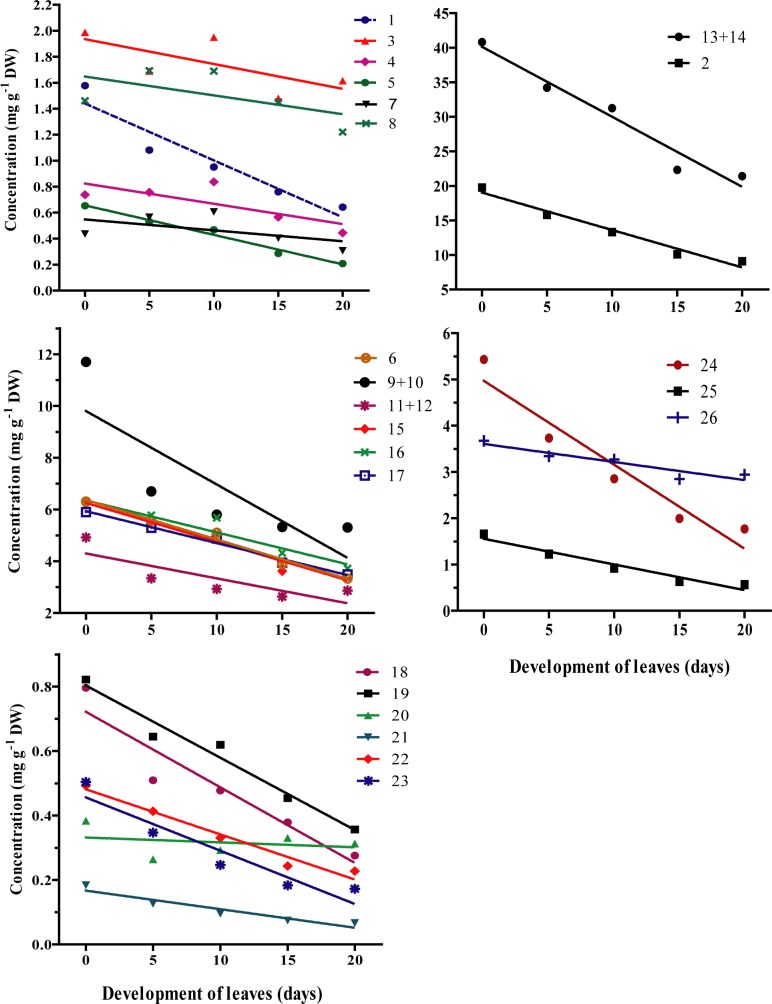
Dynamic of concentrations of phenolics and nitrogen in *Betula pendula* leaves after its opening. Regression lines between concentrations and the time of development are presented for each compound (or sum of compounds) numbered according to the numbering in Table 1.

The concentration of nitrogen as well as the concentrations of most low-molecular weight phenolics (i.e hydroxycinnamic acid derivative # 1 and #3; 3,4'-dihydroxypropiophenone-3-β-D- glucopyranoside; coumaroylquinic acid isomer; all glycosides of quercetin; all glycosides of kaempferol; aclycones of kaempferol and apigenin; monomethyl and dimethyl ethers tetrahydroxylated flavone; trimethyl ether pentahydroxylated flavone) were decreased with the maturation of *B*. *pendula* leaves (Table 1, Fig 2).

**Table 1:** The statistical analysis of the leaf chemistry dynamics during spring development of *Betula pendula* leaves (values are presented in Fig 2) carried out with regression analysis.

Nr	Compounds	*r* ^*2*^	*P*	Regression equation
1	Hydroxycinnamic acid derivative # 1	0.910	0.012	y = 1.440–0.044*x
2	3.4'-Dihydroxypropiophenone-3-β-D- glucopyranoside (DHPPG)	0.969	0.002	y = 19.032–0.542*x
3	Chlorogenic acid	0.477	0.196	y = 1.935–0.0191*x
4	Hydroxycinnamic acid derivative # 2	0.596	0.126	y = 0.824–0.016*x
5	Hydroxycinnamic acid derivative # 3	0.981	0.001	y = 0.656–0.023*x
6	Coumaroylquinic acid isomer	0.982	0.001	y = 6.368–0.153*x
7	Feruloylquinic acid	0.296	0.343	y = 0.547–0.008*x
8	Coumaroylquinic acid derivative	0.337	0.305	y = 1.647–0.014*x
9+10	Myricetin-3- glucuronide+Myricetin-3-hexoside	0.685	0.084	y = 9.804–0.284*x
11+12	Myricetin-3-rhamnoside+ Myricetin-3-arabinoside	0.684	0.084	y = 4.300–0.096*x
13+14	Quercetin-3- glucuronide+Quercetin-3- hexoside	0.956	0.004	y = 40.141–1.014*x
15	Quercetin-3- arabinoside#1*	0.960	0.004	y = 6.257–0.150*x
16	Quercetin-3- arabinoside#2*	0.893	0.015	y = 6.350–0.123*x
17	Quercetin-3- rhamnoside	0.986	<0.001	y = 5.935–0.123*x
18	Kaempferol-3-arabinoside	0.900	0.014	y = 0.722–0.023*x
19	Kaempferol-3- rhamnoside	0.967	0.003	y = 0.803–0.022*x
20	Quercetin	0.070	0.666	y = 0.332–0.002*x
21	Kaempferol	0.905	0.013	y = 0.167–0.006*x
22	Apigenin	0.966	0.003	y = 0.481–0.014*x
23	Monomethyl ether tetrahydroxylated flavone	0.898	0.014	y = 0.456–0.016*x
24	Dimethyl ether tetrahydroxylated flavone	0.925	0.009	y = 4.969–0.181*x
25	Trimethyl ether pentahydroxylated flavone	0.949	0.005	y = 1.555–0.055*x
26	Nitrogen	0.874	<0.001	y = 3.606–0.039*x

*arabinopyranoside or arabinofuranoside

### The effect of phenological mismatch on gypsy moth fitness

**Fig 3:**
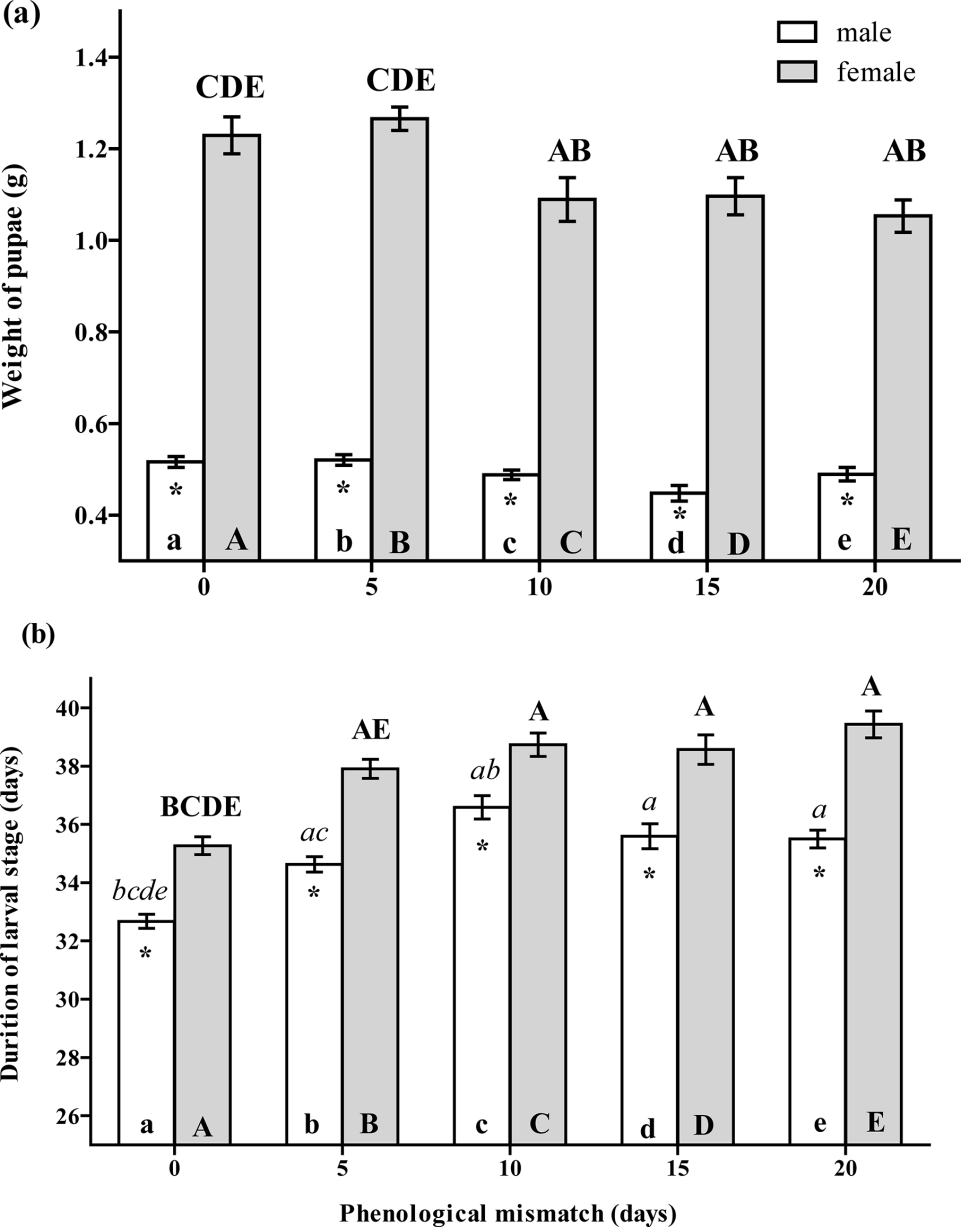
The effect of mismatch in the egg hatch of *L*. *dispar* on (a) the weight of forming pupae (mean±SE), and on (b) the speed of larvae development (mean±SE). The data were pair-wise compared by the post hoc Fisher LSD procedure. The letters above the bar mean the significant differences (at P<0.05) to be compared with the bars abbreviated by the same letters within the bar. Uppercase letters mark the differences within the female group while lowercase letters mark the differences within the male group. The asterisk means the significant differences at *P*≤0.05 between males and females within one point of mismatch.

Both of the tested factors, sex and asynchrony, significantly affected the pupal weight (*F*_1,4_ = 340.8, *P*<0.001 sex, *F*_4,15_ = 6.458, *P* = 0.003 hatching date) and the rate of larvae development (*F*_1,19_ = 104.3, *P<*0.001 sex, *F*_4,16_ = 21.94, *P*<0.001 hatching date), but a significant interaction between these factors was found only for pupal weight (*F*_4,324_ = 3.857, *P* = 0.004) not for development rate (*F*_4,19_ = 1.025, *P* = 0.420). The female pupal weight was significantly decreased when the larval hatching was mismatched 10 days or more: no significant effect was observed after a five-day mismatch in the hatching (Fig 3A). The larval development rate of both sexes, in turn, was decreased after a five-day mismatch in the hatching (Fig 3B). The female larvae were markedly larger than the male larvae and their development time was also longer (Fig 3A and 3B).

**Fig 4:**
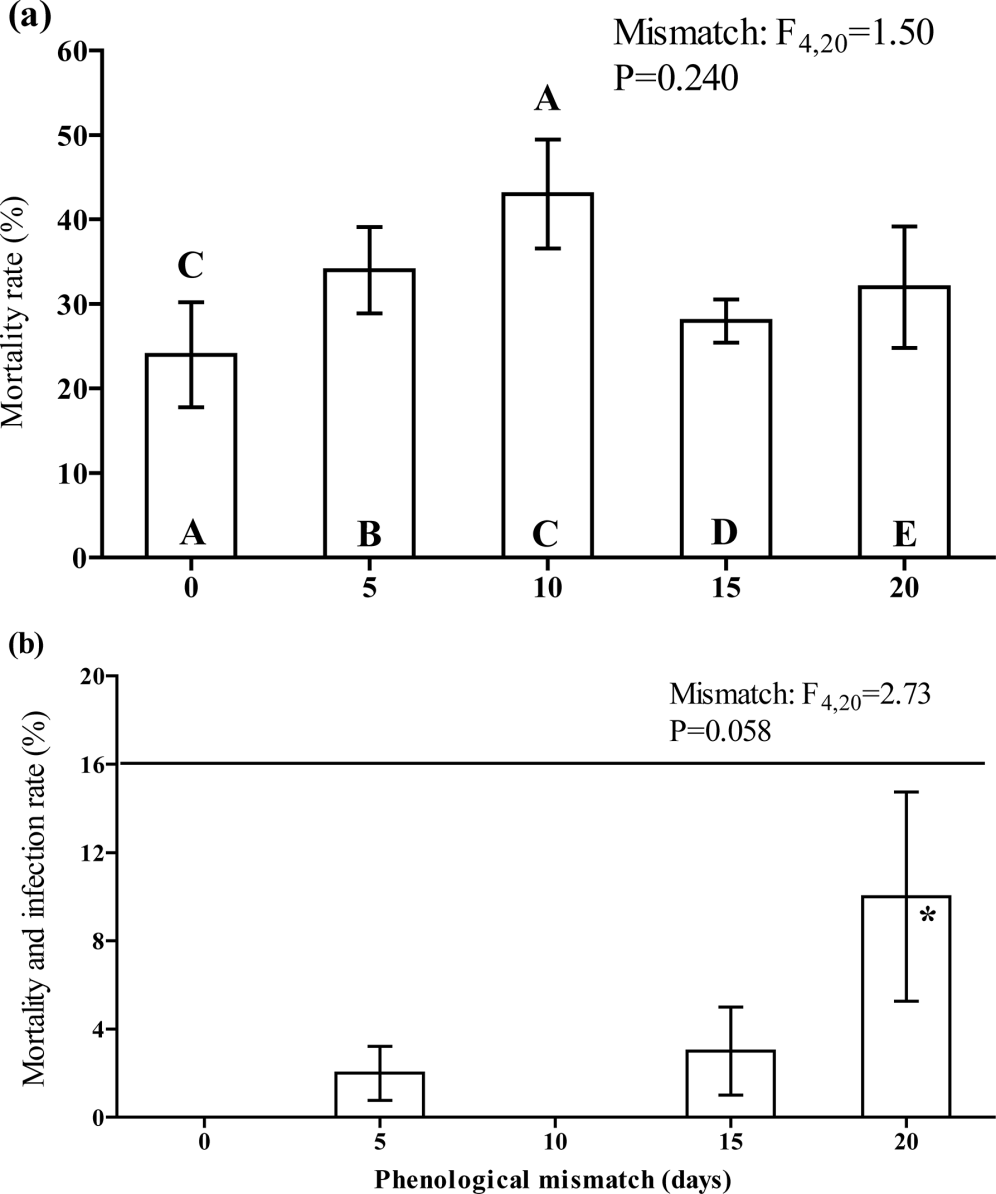
Effect of mismatch in egg hatch of *L*. *dispar* (a) on general mortality rate of insects until adults (mean±SE), and (b) on mortality rate induced by covert LdMNPV (mean±SE). The black line shows the percentage of viral DNA-positive eggs. The ANOVA statistics is presented within the figure. The letters above the bar mean the significant differences (at P<0.05) to be compared with the bars abbreviated by the same letters within the bar compared in detail by the Fisher LSD-method.

The total mortality rate of insects was not significantly affected by mismatch but there was a noticeable trend of the mortality rate increasing at 10 days of mismatch (Fig 4A). However, the etiology of mortality reveals the effect of mismatch on the mortality rate induced by covert LdMNPV with the maximal value at 20-days' mismatch (Fig 4B). PCR analysis revealed that 16% of the eggs of the stock of the *L*. *dispar* cohort contained the cover virus above the detection limit (Fig 4B, the black line). Thus, the maximal mismatch in the hatching induced viral pathogenesis in more than half of the larvae that carried a covert viral infection (the mortality rate in Fig 4B to compare with the black line).

### The effect of phenological mismatch on *L*. *dispar* immune traits

**Fig 5:**
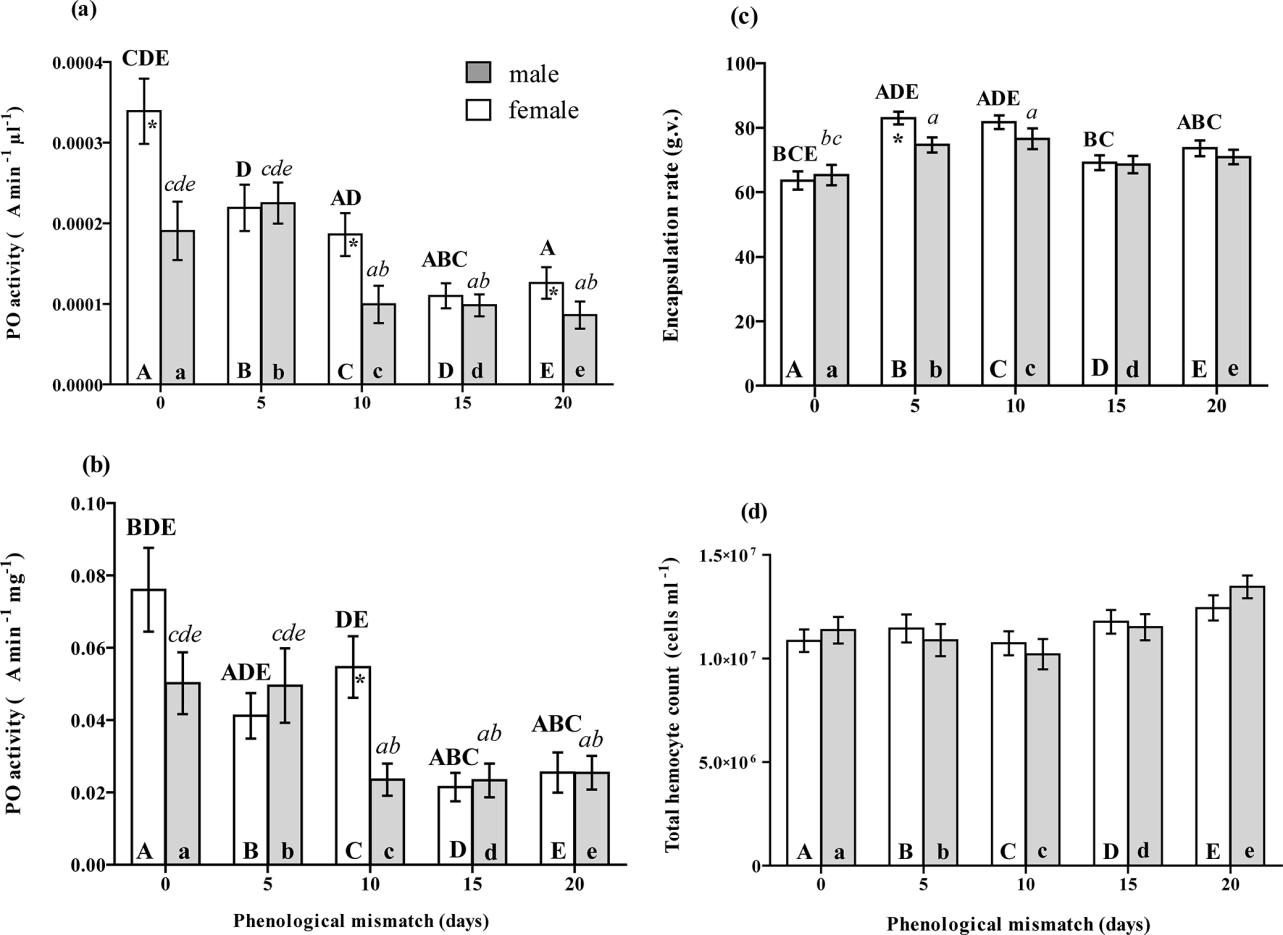
The effect of mismatch in the egg hatch of *L*. *dispar* on (a) the phenoloxidase activity in the plasma of fourth instar larvae, measured as ΔA_490_ min^-1^μl^-1^ plasma (mean±SE), on (b) the phenoloxidase activity in the plasma of fourth instar larvae, measured as ΔA_490_ min^-1^ mg^-1^ protein (mean±SE), on (c) the encapsulation of the nylon implant inserted into the hemocoel of fourth instar larvae (mean±SE), and on (d) the total hemocytes count in the haemolymph of fourth instar larvae (mean±SE). The data were pair-wise compared by the post hoc Fisher LSD procedure. The letters above the bar mean the significant differences (at P<0.05) to be compared with the bars abbreviated by the same letters within the bar. Uppercase letters mark the differences within the female group while lowercase letters mark the differences within the male group. The asterisk means the significant differences at *P*≤0.05 between males and females within one point of mismatch.

Phenoloxidase (PO) activity in larval plasma was significantly decreased (*F*_4,36_ = 9.223, *P*<0.001 measured as ΔA μl^-1^ plasma; *F*_4,35_ = 6.672, *P* = 0.001 measured as ΔA mg^-1^ protein) when the hatching of larvae was mismatched (Fig 5A and 5B). This decrease was linearly mismatch-dependent: *n* = 5; *r* ^*2*^ = 0.88, b = -0.0232±0.0050, *P* = 0.018 for females, *n* = 5, *r* ^*2*^ = 0.77, b = -0,021±0,0068, *P* = 0.053, for males, where ΔA μl^-1^ plasma, *n* = 5 *r* ^*2*^ = 0.73, b = -0.0247±0.0086, *P* = 0.064 for females, *n* = 5 *r* ^*2*^ = 0.70, b = -0.0184± 0.0070, *P* = 0.078 where ΔA mg^-1^ protein. Females possessed significantly higher PO activity compared to that of males when activity was measured as ΔA μl^-1^ plasma (*F*_1,36_ = 7.573, *P* = 0.009 calculated as ΔA μl^-1^ plasma, *F*_1,36_ = 2.126, *P* = 0.154 calculated as ΔA mg^-1^ protein).

In contrast to PO activity, the encapsulation rates of both female and male larvae were increased (*F*_*4*,*16*_ = 8.963, *P* = 0.001) when the hatching was mismatched for 5 and 10 days as compared to the synchronized larvae, but this effect was relaxed when the mismatch was increased to 15 and 20 days (Fig 5C). Sex had no significant effect on the encapsulation rate (*F*_1,7_ = 1.223, *P* = 0.304). However, pairwise comparison shows the significant difference between both sexes in the group of 5 days mismatched (Fig 5C). Neither sex (*F*_1,16_ = 0.003, *P* = 0.954) nor hatching date (*F*_4,20_ = 1.503, *P* = 0.239) had a significant effect on the THC of larval haemolymph (Fig 5D).

### The effect of phenological mismatch on *L*. *dispar* susceptibility to exogenous administration by LdMNPV

**Fig 6:**
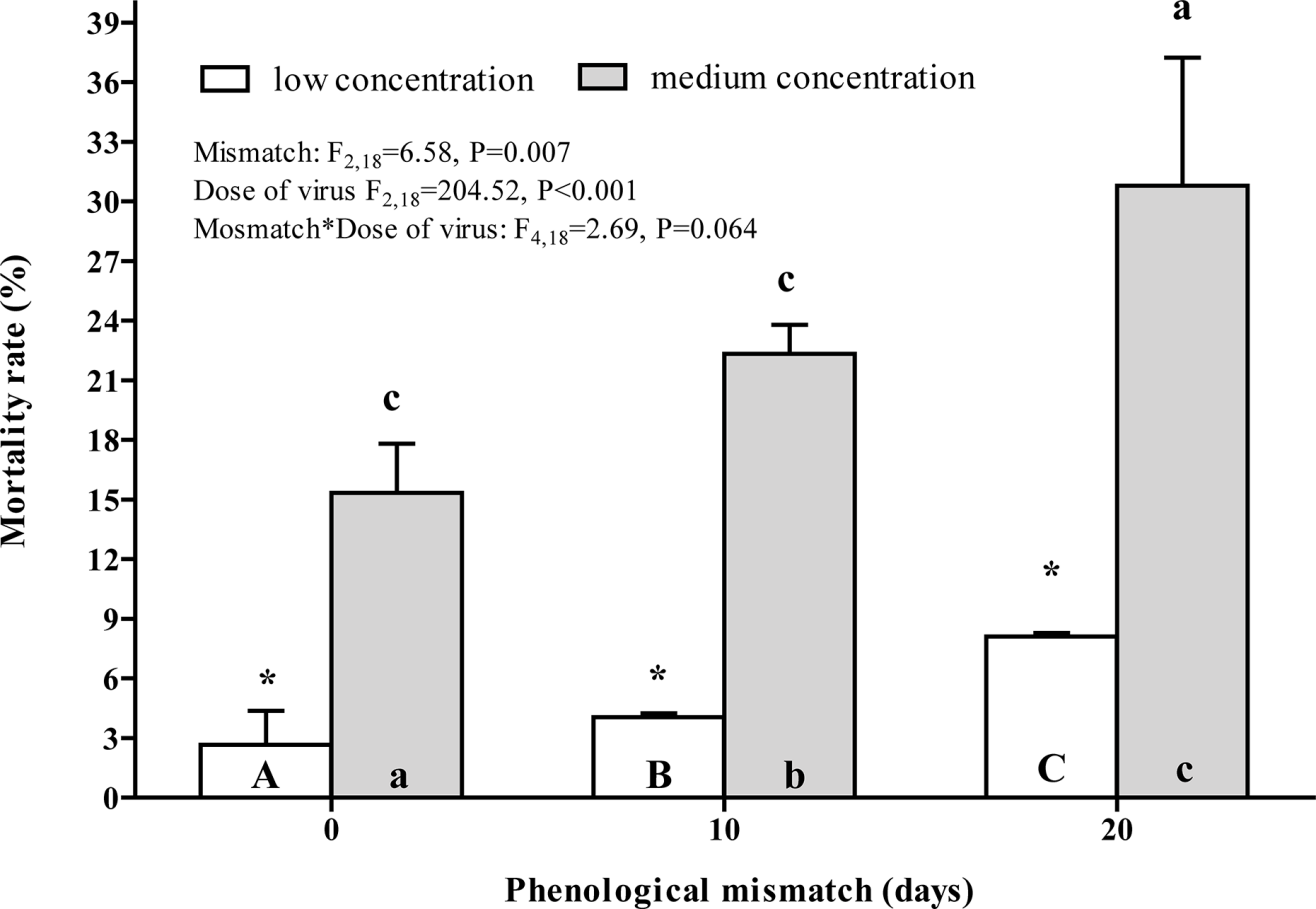
The effect of mismatch in the egg hatch of *L*. *dispar* on the susceptibility of the fourth instar caterpillars to exogenous administration by low (empty bars) and medium (filled bars) concentrations of nucleopolyhedrovirus. The ANOVA statistics is presented within the figure. A more detailed comparison was made using the post hoc Fisher LSD procedure. The letters above the bar mean the significant differences (at P<0.05) to be compared with the bars abbreviated by the same letters within the bar. Uppercase letters mark the differences within the low concentration group while the lowercase letters mark the differences within the medium concentrations group. Asterisks mean the differences (at P<0.05) between doses within one point of mismatch.

The mortality rate of larvae after per os LdMNPV infection displayed the same trend for both tested concentrations of virus: virus-induced mortality was higher in the maximally mismatched group of larvae (Fig 6).

## Discussion

For the first time we were able to demonstrate that the phenological mismatch between *L*. *dispar* larval hatching and host tree budding severely affects larval immunity and the ability to activate covert baculoviral disease. The larval mortality of *L*. *dispar* infected by LdMNPV was increased when larval hatching was delayed. In addition, the pupal weights of female moths were markedly decreased and the development rates of both sexes decreased.

An interesting finding in our present study is that a significant phenological mismatch led to an increase in the activation of the covert virus in the *L*. *dispar* larvae. Since our experimental design excludes the horizontal or transoval transmission of baculovirus, only an internal virus could be the reason of LdMNPV-induced larval mortality. In spite of the comparatively low rate of exogenous virus-induced mortality in the studied population (10% at maximal phenological mismatch) we found that ca. 62% of larvae carrying the covert virus died from the activated viral disease at the 20-day mismatch. Consequently, if covert LdMNPV persisted above a detectable level in most individuals of the *L*. *dispar* population, as it sometimes occurs in the Siberian region [47–48] or in other regions for other Lepidopterans [49–50], the asynchrony would dramatically affect the population size of the spring feeding moths through the activation of the covert nucleoplyhedroviruses. Moreover, since a mismatch also increases the susceptibility of larvae to exogenous infection by LdMNPV, as shown in the present study, the investment of the horizontal transmission of LdMNPV could also be increased in the moth population hatched with delay. Both these findings, together with the long development of the larvae (the vulnerable stage for insects in relation to baculovirus diseases, [51]) could explain the source of large scale epizootics induced by baculoviruses in outbreaks of gypsy moth and possibly in other species.

A recent immunological study has clearly shown that variation in *L*. *dispar* immune function (including PO activating cascade)determined by the ageing of larvae within an instar affects the host’s resistance to the administration of LdMNPV [52]. The antiviral capability of PO against different viruses including baculovirus was also demonstrated in other Lepidopteran species, *Heliothis virescens* [53–54]. Finally, Zhao and co-authors recently and clearly demonstrated the antiviral effect of 5,6-dihydroxyindole, a reactive compound generated by phenoloxidase during the insect immune response [55]. Thus, we suppose that the reduction in the baseline activity of such an immune parameter as the PO activity in the plasma of larvae hemolymph under the effect of phenological mismatch is directly associated with the increased level of mortality in insects induced by covert virus infection. This decrease in PO activity is facilitated by the decline in nitrogen content in the leaves of maturing leaves *B*. *pendula*. The decline in the concentration of nitrogen reflects the decrease in the concentrations of amino acids (mainly protein-bounded) that was demonstrated in the closely-related species of birch (*Betula pubescens* subsp. *czerepanovii* (Orlova) Hämet-Ahti) during the development of leaves within season [6, 56]. On the other hand, by modifying the protein content in the diet of *Spodoptera littoralis*, Lee and co-authors clearly demonstrated the importance of proteins for the larvae PO activity and other immunological parameters, as well as for resistance to baculovirus infection [19]. Thus, the observed decline in the protein diet (estimated as nitrogen content) of mismatched larvae in our study might be the main reason for the decrease of PO activity in the plasma of haemolymph of *L*. *dispar* larvae and the following decrease of their antiviral resistance.

There is also another possible mechanism of asynchrony the triggered alteration of larval susceptibility to exogenous LdMNPV. It is the direct interaction of phenolics with the virus within the midgut lumen during the dissolving of polyhedrons (i.e. [57–58, 32]). It is unlikely that in our study that hydrolysable tannins were involved in the increase of viral potency (as was shown by Keating and co-authors [58] for other plant species) because it almost disappears in the silver birch leaves immediately after its expansion [8] and remains at a very low level later [44]. However, the decreased low-molecular phenolics in matured leaves might possess the negative feedback with viral potency through the decrease of the direct damaging of released virions within the larval midgut.

We have also shown that mismatch negatively affects the fitness of insects, as has been shown in many other studies [1–5, 42]. Most of the studied phenolics of silver birch leaves, including the compounds toxic for Lepidopteran such as dihydroxypropiophenone-3-β-D- glucopyranoside or lipophilic aglycones [59–61] decreased during leaf maturation as well. Two possible explanations may account for this observation. First, not only secondary compounds but the ratio between allelochemicals and nutrients in host plant leaves is important for the maximally successful development of *L*. *dispar* individuals. Second, the dynamic of other secondary compounds is possibly more important for the phenologically driven decrease of insects' fitness. In particular, our recent study shows the importance of lypophilic triterpenoids which are the component of *B*. *pendula* chemical defence [24].

Thereby our findings partially explain the scenario of the fluctuation in the *L*. *dispar* population size when weather conditions provoke or do not provoke the phenological mismatch between the host plant and the herbivore. It is known that the temperature threshold of the spring development of *B*. *pendula* is close to -1…2°C [62]. while the temperature threshold of the spring development of *L*. *dispar* before hatching is higher (about 6°C) [63]. Consequently, cold weather in spring will generate the lag of insect development within eggs from the development of birch buds. During several such unfavourable years, the population of herbivores is retained at a low level because insects are more likely to feed on the maturing leaves of the host plant. Low dietary intake leads to prolonged development, decreased immune function, and resistance to baculovirus (both endogenous and exogenous), as well decreased female fecundity. If the weather conditions in spring are favourable (warm weather), any lag in insect maturation attributable to leaf development will be minimal and all of these parameters will be modified in the opposite direction. The quality of consumed leaves will be enough to increase the investment in both the innate immunity and the fitness of larvae which then allows for a decrease in the pressure from parasites and to a significant increase in the coefficient of population reproduction. Two successive years of maximal realisation of a population’s biotic potential could lead to an outbreak of the pest. If the weather conditions are changed again and become unfavourable, the situation will be repeated as described above. The increased density of specialized pathogens after a peak in the host population density will assist a faster decline in the folivore population density and provoke viral epizootics if the level of the harboured virus in the host population is high. We do not assert that this is the only mechanism (shown under laboratory conditions) that operates in natural populations of *L*. *dispar*. For example, one of the effective adaptations of insects, evolved during phylogenesis to overcome the effect of asynchrony, is the natural variation in gypsy moth larvae emergence, even amongst those hatching within the same clutch. Moreover, the rate of endogenous virus in insect populations is significantly varied and is often kept at a low level (reviewed in [64]). However, the current study highlights the importance of herbivore—baculovirus interaction in those cases when most larvae of the gypsy moth population will hatch out together (for example, during a long period of warm weather, or vice versa, amid a long period of cool weather following warm weather in the spring) and especially in those cases when the endogenous baculovirus persists in most individuals of the *L*. *dispar* that occur in the gypsy moth population [25, 47–48, 65]. This study highlights the respective importance of theinnate immune functions and the fitness of insects both of which are driven by host plant development within season.

## Supporting Information

S1 Text Detailed methods used in this study. (DOC)
