# Supplementary material for: Asynchrony between Host Plant and Insects-Defoliator within a Tritrophic System: The Role of Herbivore Innate Immunity
Source: PLoS One. 2015 Jun 26;10(6):e0130988. doi: 10.1371/journal.pone.0130988 (PMC4482610; doi:10.1371/journal.pone.0130988)
Supplement: S1 Text — (DOC) [file pone.0130988.s001.doc]

**Immune assay**

To collect haemolymph, the cuticle of each caterpillar was pierced with a thin needle under the penultimate proleg. About 20–25 μl of hemolymph from each larva were collected, and then placed into two cooled 1.5 ml Eppendorf tubes. One aliquot (4.5 μl) was mixed with 20 μl of cooled anticoagulant with phenylthiourea to study THC. THC was immediately measured in a hemocytometer and recorded as the number of haemocytes per 1 ml of haemolymph. The other aliquot of haemolymph was centrifuged at 500×g and +4°C for 10 min, and the supernatant was used to estimate PO activity, and protein concentration. PO activity was measured by using L-dopa as a substrate. Ten μl of supernatant were mixed with 500 μl of L-dopa in phosphate buffer, pH 7.2 (concentration 2 mg/ml solution). After incubation for 1 hr at 28°C, PO activity was measured at 490 nm with an Agilent 8453 UV-visible spectroscopy system. Haemolymph protein levels were measured by methods described by Bradford (1976), with a standard curve created from a bovine serum albumin standard. Phenoloxidase activity was measured in units of transmission density (ΔA) of the incubation mixture during the reaction per 1 min and 1 mg of protein.

The encapsulation response was measured as the degree of melanization of a nylon monofilament implant that was inserted into the hemocoel of the larvae. To measure the encapsulation response, we inserted the monofilament into insects of the same group immediately after haemolymph collection. A 2 mm piece was inserted into the larva’s body cavity through the hole made for haemolymph collection. The implants were dissected from the body cavity after 3 hr of exposure and then photographed in a black-and-white format from three perspectives. The degree of the melanization was quantified by using Image Pro software by first measuring the coloration—gray value (g.v.) of all areas on each implant, and then comparing these values with that of an unused implant. Larvae remained alive after the above described procedure and that allowed us to record its sex. Indeed, each larva was reared individually to adulthood once the immune measurement had been taken. The sex was recorded based upon the structure of the antenna.

**Insect infection by LDMNPV**

Larvae assigned for the infection by LDMNPV were used for the peroral inoculation after they reached fourth instar. The Tatarskiy strain of LdMNPV (a strain held in the laboratory collection of the Institute of Systematics and Ecology of Animals at the Siberian Branch of the Russian Academy of Sciences) was used for the inoculation. This virus strain was previously isolated from the L. dispar population inhabiting Western Siberia. The leaves of each B. pendula tree (30 leaves per tree) first were washed with sterile distilled water, as described in the procedure on larvae rearing, to reduce the chance of contamination by any external entomopathogenic agents. Then, after the drying of the washed leaves they were sprayed with a water-virus suspension (50 ml of each concentration per leaves from each tree). After the leaves were dried at room temperature in the shade they were presented to the appropriate groups of larvae (corresponding to the trees from whence they came). After the larvae had consumed all the leaves, they were fed on fresh uninfected leaves. Then, larvae were checked daily until pupation and following molting to adulthood. The susceptibility of the larvae to LdMNPV was calculated as the total percentage of insects deaths from viral disease during ontogenesis.

**The details of PCR analysis of *L.dispar* eggs for detecting of covert LdMNPV**

After sterilization, individual eggs were crushed and ground in 50 µL TE (10mM Tris, 1mM EDTA, pH 8). One mL of carbonate buffer pH 10.8 (0.1 M Na2CO3, 0.17 M NaCl, 0.01 M EDTA) was added to the suspension of the crushed eggs and this mixture was incubated at room temperature for 30 min. The suspension was then incubated with SDS and proteinase K (final concentrations are 0.5% and 0.05 mg mL-1 respectively) for 3 h at 60^o^C in shaking. Before the reaction the pH was reduced from 10.8 to 8 by adding 10% acetic acid. Afterwards, DNA extraction was done according to the methods described by Castro et al. (2009).

Primers for the PCR where selected corresponding to the LdMNPV polyhedrin gene sequence [1]. The LdMNPV primers where: 5'-TCAGAAACTCACTCTCTTCAAAGAG-3' (forward) and 5'-CACGTACACGATGGGCTTGTAG-3' (reverse). The ampliﬁcation reactions were performed in a final volume of 25 µL contained 2.5 µL of 10x buffer for Taq DNA polymerase (0.1 M Tris HCl pH 9, 0.5 M KCl, 1% Triton X-100, H2O), 1.6 mM MgCl2, 0.2 mM of each of four dNTPs, 0.2 µM of both the forward and reverse primer, 1 unit of Taq DNA polymerase, 7 µL of studied samples and H2O for final volume. The cycling parameters for PCR were as follows: cycle 1 - 2 min at 95 °C; cycle 2, first step, 1 min at 95 °C, second step, 1 min at 52 °C, third step, 1 min at 72 °C (45 cycles); the last cycle - 5 min at 72 °C. Amplification was accomplished with a DNA Thermal Cycler “BIS” M-111 (DNK Tekhnologiya, Moscow). Following amplification, the PCR products were analyzed by a 1% agarose gel electrophoresis in 1x TAE buffer.

**The HPLC conditions**

The MS was used with the electrospray ionization (ESI) source. MS conditions were as follows: Vcap 4000 V; nebulizer pressure 1.6 bar; drying gas (N2) flow 8 L min-1; drying gas temperature 230 °C; negative and positive scan in the range m/z = 80–3000. Separation was achieved on an Eclipse XDB-C8 (4.6 × 150 mm, 5 μm) column by use of a binary solvent system consisting of 0.1% CF3COOH in water (DAD) or 2% HCOOH (MSD) (A) and methanol (B). The gradient (% B) was linear from 2 to 95 % in 30 min, the HPLC runs were monitored at 280 nm for 3,4'-dihydroxypropiophenone-3-β-D-glucopyranoside (DHPPG), at 320 nm for cinnamic acids, and at 360 nm for flavonoids [2].

References

1. Katalinic V. () High-perfomance liquid chromatographic determination of flavan fingerprint in plant extracts. J Chromatogr A. 1997; 775, 359-367.
2. Woo SD. Rapid Detection of Multiple Nucleopolyhedroviruses Using Polymerase Chain Reaction. Mol Cell. 2001; 11:334-340.
